# Pain and Attention-Deficit/Hyperactivity Disorder: A Closer Look at the Relationship

**DOI:** 10.1192/j.eurpsy.2025.1894

**Published:** 2025-08-26

**Authors:** F. Lobo, A. M. Fernandes, C. Eusébio, F. Agostinho, L. Figueiredo, M. J. Amaral, R. Avelar, M. J. Heitor

**Affiliations:** 1 Departamento de Psiquiatria e Saúde Mental, Hospital Beatriz Ângelo, Loures, Portugal

## Abstract

**Introduction:**

Attention-Deficit/Hyperactivity Disorder (ADHD) is a prevalent neurodevelopmental disorder characterized by symptoms of inattention, hyperactivity, and impulsivity. Recent research highlights a significant comorbidity between ADHD and pain, suggesting that individuals with ADHD may experience altered pain perception and a higher prevalence of pain conditions.

**Objectives:**

This review aims to explore the possible link between pain and ADHD, specifically examining the relationship between ADHD and pain perception, the effects of methylphenidate (MPH) on pain thresholds, and the potential underlying mechanism connecting these two conditions.

**Methods:**

A non-systematic review of the literature was conducted, focusing on key studies published in the last 15 years. The search terms included “pain,” “ADHD,” and “methylphenidate.”

**Results:**

Based on this review, several key findings emerged:

- **Dopamine’s Role in Pain:** Accumulating data suggest that dopamine is implicated in pain processing. Many regions of the CNS involved in pain processing have high dopamine receptor density, whose activation can be analgesic.
- **Increased Prevalence of Chronic Pain:** Individuals with ADHD show a higher prevalence of chronic pain conditions, indicating a significant comorbidity between ADHD and pain.
- **Altered Pain Perception:** Individuals with ADHD are more likely to exhibit lower pain thresholds and increased pain sensitivity, particularly in untreated individuals.
- **Impact of Methylphenidate:** Methylphenidate, a common treatment for ADHD, partially reverses altered pain responses, suggesting its potential role in normalizing pain perception through dopaminergic modulation.
- **Neuroinflammation as a Link:** Neuroinflammation has been suggested as a potential factor linking ADHD and pain, particularly through dopaminergic dysregulation.

**Conclusions:**

This review underscores the need for increased awareness of the pain-ADHD comorbidity. Understanding altered pain perception in ADHD is crucial for improving patient care and developing targeted treatments. While current evidence suggests treatments like methylphenidate may help modulate pain sensitivity, further research is essential to clarify the mechanisms and establish guidelines for managing pain in ADHD patients.

**Disclosure of Interest:**

None Declared

